# Balancing competing priorities: Quantity versus quality within a routine, voluntary medical male circumcision program operating at scale in Zimbabwe

**DOI:** 10.1371/journal.pone.0240425

**Published:** 2020-10-13

**Authors:** Caryl Feldacker, Vernon Murenje, Batsirai Makunike-Chikwinya, Joseph Hove, Tinashe Munyaradzi, Phiona Marongwe, Shirish Balachandra, John Mandisarisa, Marrianne Holec, Sinokuthemba Xaba, Vuyelwa Sidile-Chitimbire, Mufuta Tshimanga, Scott Barnhart

**Affiliations:** 1 International Training and Education Center for Health (I-TECH), Seattle, WA, United States of America; 2 Department of Global Health, University of Washington, Seattle, WA, United States of America; 3 International Training and Education Center for Health (I-TECH), Harare, Zimbabwe; 4 Zimbabwe Association of Church-related Hospitals (ZACH), Harare, Zimbabwe; 5 Zimbabwe Community Health Intervention Project (ZICHIRE), Harare, Zimbabwe; 6 United States Centers for Disease Control and Prevention, Division of Global HIV & TB, Harare, Zimbabwe; 7 Ministry of Health and Child Care, Harare, Zimbabwe; 8 Department of Medicine, University of Washington, Seattle, WA, United States of America; University of Ghana College of Health Sciences, GHANA

## Abstract

**Background:**

Since 2013, the ZAZIC consortium supported the Zimbabwe Ministry of Health and Child Care (MOHCC) to implement a high quality, integrated voluntary medical male circumcision (VMMC) program in 13 districts. With the aim of significantly lowering global HIV rates, prevention programs like VMMC make every effort to achieve ambitious targets at an increasingly reduced cost. This has the potential to threaten VMMC program quality. Two measures of program quality are follow-up and adverse event (AE) rates. To inform further VMMC program improvement, ZAZIC conducted a quality assurance (QA) activity to assess if pressure to do more with less influenced program quality.

**Methods:**

Key informant interviews (KIIs) were conducted at 9 sites with 7 site-based VMMC program officers and 9 ZAZIC roving team members. Confidentiality was ensured to encourage candid conversation on adherence to VMMC standards, methods to increase productivity, challenges to target achievement, and suggestions for program modification. Interviews were recorded, transcribed and analyzed using Atlas.ti 6.

**Results:**

VMMC teams work long hours in diverse community settings to reach ambitious targets. Rotating, large teams of trained VMMC providers ensures meeting demand. Service providers prioritize VMMC safety procedures and implement additional QA measures to prevent AEs among all clients, especially minors. However, KIs noted three areas where pressure for increased numbers of clients diminished adherence to VMMC safety standards. For pre- and post-operative counselling, MC teams may combine individual and group sessions to reach more people, potentially reducing client understanding of critical wound care instructions. Second, key infection control practices may be compromised (handwashing, scrubbing techniques, and preoperative client preparation) to speed MC procedures. Lastly, pressure for client numbers may reduce prioritization of patient follow-up, while client-perceived stigma may reduce care-seeking. Although AEs appear well managed, delays in AE identification and lack of consistent AE reporting compromise program quality.

**Conclusion:**

In pursuit of ambitious targets, healthcare workers may compromise quality of MC services. Although risk to patients may appear minimal, careful consideration of the realities and risks of ambitious target setting by donors, ministries, and implementing partners could help to ensure that client safety and program quality is consistently prioritized over productivity.

## Introduction

Since research found that voluntary medical male circumcision (VMMC) reduced the risk of female-to-male HIV-1 transmission by up to 60% [1–3], nearly 19 million VMMCs were performed across 14 sub-Saharan African (SSA) countries between 2008–2017 [4]. As VMMC is a priority HIV prevention intervention across SSA, in 2016, the United Nations set an annual target of 5 million VMMCs to reach 25 million men in SSA by 2021 [5]. However, healthcare constraints threaten quality and pace of current VMMC scale-up [6–8], including in Zimbabwe where pervasive economic constraints affect public services [9], including healthcare [10]. As failure to reach VMMC targets means fewer infections averted [11–13], there has been a global push to reach VMMC targets quickly. However, ambitious targets may be incompatible with quality care provision. The quality of VMMC care can be measured by the rate of adverse events (AEs) and post-operative follow-up visit attendance [14]. In South Africa, strained human resources contribute to pervasive weakness in AE detection, diminishing VMMC patient care [15]. Similar problems are likely occurring in other SSA countries as the push to reach 80% VMMC coverage expands access via outreach (primary healthcare clinics, schools, communities). In these more rural and remote settings, the burden of VMMC services falls on under-resourced clinics and providers [16,17]. Coupled with client challenges of high transportation costs, long wait times, and limited service availability [18,19], VMMC quality may suffer.

ZAZIC, a consortium founded in 2013 as a collaboration between International Training and Education Center for Health (I-TECH), University of Zimbabwe College of Health Sciences Clinical Trials Research Centre (UZCHS-CTRC), Zimbabwe Association of Church related Hospitals (ZACH) and Zimbabwe Community Health Intervention Research Project (ZICHIRE), cooperates with Zimbabwe’s Ministry of Health and Child Care (MoHCC) to implement an integrated VMMC program in 13 districts in Zimbabwe. From March, 2013 to September, 2019, ZAZIC conducted over 450,000 VMMCs; the reported moderate and severe AE rate was 0.3% [20]. Ensuring continuous quality service provision is a critical component of ZAZIC’s VMMC program. ZAZIC implements routine monitoring and evaluation (M&E) activities to identify and address weaknesses in data collection, data reporting, and data use. Reported adherence to follow-up visits, another indicator of program quality [21], was good with routine data indicating 90% of men attending at least one review visit within 14 days of procedure. However, ZAZIC investigation focused on active surveillance of AEs and found higher AE rates (~4%; range 1–8%) and, as part of the study, observed lower follow-up than reported [22]. As ZAZIC strives to achieve high VMMC productivity targets, early signs of weakness in quality service delivery require immediate action to maintain its dedication to both patient safety and program performance.

This paper explores the responses of ZAZIC and MoHCC VMMC providers to the question of whether the drive to maintain momentum and prioritize productivity may compromise VMMC quality. Over two weeks, key informant interviews were conducted with District Medical Officers (DMOs) and ZAZIC clinical staff from roving teams as part of routine quality assurance activities aiming to illuminate impressive VMMC productivity data and gain insights into the realities of field implementation. Our specific objectives were to: 1) gain understanding of the quality versus quantity tradeoffs in VMMC implementation; 2) gain understanding of how increased productivity potentially affects patient safety; and 3) use the results to inform scale up or modification of the national VMMC program in Zimbabwe to maintain productivity while prioritizing patient safety. Better understanding of the tradeoffs between quantity and quality of the VMMC program could help modify and strengthen the national program in Zimbabwe.

### Routine ZAZIC VMMC program

The locally-led ZAZIC consortium, was established in 2013 to build health system capacity through integrated VMMC program delivery in partnership with the Zimbabwe MoHCC. All VMMC guidelines are followed, including safety standards for the identification, management, and reporting of AEs and conducting routine, in-person follow-up at days 2, 7 and 42 after the VMMC procedures [23]. In the first 5-year cooperative agreement, ZAZIC improved productivity each year of operation, contributing 276,242 VMMCs between March 2013 and March 2018 with an average of 2000 VMMCs/week and a reported AE rate of under 1%. Overall VMMC performance surpassed 450,000 by the end of September, 2019. In ZAZIC-supported districts, MoHCC teams performed the vast majority of VMMCs supplemented by ZAZIC’s mobile teams (roving teams) who augmented site-based capacity when needed, demonstrating a successful, blended implementation model. Each year, ZAZIC expanded access to VMMC services and reached or exceeded all VMMC targets. During this period, targets tripled from 40,800 VMMCs to 125,523 VMMCs with a reduced unit expenditure (UE) of $104.03 to $93.84. The UE was set annually by USG with limited input by implementing partners. ZAZIC leveraged its local faith-based, private and MoHCC partnerships to increase VMMC productivity using the most effective mix of static, outreach, and mobile sites. ZAZIC employed differentiated service delivery, adapting the model based on local contextual factors including MoHCC capacity, population distributions, and social/cultural factors. In order to ensure buy-in and human resource commitment, I-TECH implemented a performance-based financing (PBF) model, described in detail previously [24]. Additionally, ZAZIC implemented comprehensive training (refresher and new) for VMMC teams (clinicians and non-clinicians) to ensure high quality clinical care, adherence to guidelines, improved understanding of reporting requirements, and quality data collection. Demand creation was integral to the program, utilizing human-centered design approaches to tailor localized demand creation efforts to meet targets for VMMC. Establishing and training community-based outreach teams to develop and implement culturally-appropriate motivational messages, activities, and strategies helped increase uptake [25].

## Materials and methods

### Ethics

This study was conducted with approval by the Medical Research Council of Zimbabwe (MRCZ), Research Council of Zimbabwe (RCZ), Centers for Disease Control and Prevention (CDC), and the University of Washington (UW) internal review. In accordance with federal and UW Human Subjects Division policy, we received a non-research determination for these program study activities. The protocol was reviewed in accordance with CDC human research protection procedures and was determined to be research, but CDC investigators did not interact with human subjects or have access to identifiable data or specimens for research purposes. The written informed consent process was approved as part of the ethical review and was implemented by the study team. As part of the consent procedures, all participants were informed that their participation was voluntary. Subjects were assured that their employment and participation in the VMMC program would not be affected by their study participation. Participant interviews were recorded and data transcribed and verified. The interviewer and notetaker reviewed human subject’s considerations and signed confidentiality statements. Original audio recordings were not reviewed by any member of the local research team to protect participants. No names were transcribed for analysis.

### Study preparation

To prevent possible bias and protect confidentiality, both the qualitative interviewer and notetaker, were selected based on their familiarity of the VMMC program and that they had previously worked for the ZAZIC consortium, but were not currently connected to the program or employed by ZAZIC. The study team developed, tested, and piloted a key informant interview (KII) guide in English; the guide was minimally adapted to reflect experiences from the perspectives of ZAZIC or MoHCC KIs. The interviewer and notetaker were trained by study investigators in study protocols and pre-tested instruments. Although the interviewer and notetaker were both also fluent in Shona, all KIIs were conducted in English at the request of the participants. KIIs included questions and probes on successes and weaknesses of the VMMC program in general, and then more sensitive questions on achievement of VMMC targets, patient safety, patient follow-up, provider stress, and overall program quality.

### Site selection

From all 36 ZAZIC sites, the nine most productive ZAZIC sites based on VMMCs performed for the period March 2017 to September 2017 and all eight roving team clinicians from both ZAZIC partners were purposively selected. Distance from Harare was also considered due to the limited study funds. Provincial Medical Directors and ZAZIC leadership facilitated access to the clinics/hospitals.

### Recruitment

Eligibility criteria for the KIs included: either DMO or site focal person with at least 6 months in current position; ZAZIC roving team clinician (either a physician or nurse); able and willing to complete informed consent; older than age 18; and willing to be recorded. Those not willing to participate or be recorded were excluded.

### Implementation

KIIs were conducted individually by one experienced interviewer and one experienced notetaker in private locations from 26 August to 5 September, 2019. The interviewer and notetaker, both Zimbabwean females, had 11 and 5 years qualitative research experience, respectively. Each interview was less than 1 hour in duration. No compensation was provided. Interviewees were offered a non-alcoholic drink as an appreciation for participation. Each KII was recorded with participant consent and transcribed by a third-party consultant. The interviewer and note taker verified each transcript for accuracy.

### Data analysis

Qualitative data were entered, coded and analyzed (Atlas.ti 8.0) as text documents by one external researcher to protect KI confidentiality. Text was initially coded based on the interview guide themes. Question-based codes were augmented with new themes based on the grounded theory approach [26] that applies a cyclical approach to identifying and grouping themes generated from the participants.

## Results

A total of 17 potential respondents were approached to participate in the study; one refused. Of the 16 interviewed, 7 were men. Eight ZAZIC clinicians with site-based leadership and VMMC staff from 7 roving teams (n = 9) were interviewed. Diversity was considered in selecting illustrative quotes. To protect the privacy of the small number of known VMMC providers from sites and within the partner organizations, no identifying information (gender, location, position) is provided about the source of the quote.

### Program successes

#### Achievement of ambitious VMMC targets

VMMC team participants highlighted four ways that they ensured reaching the targets. First, echoed through all KIIs, was how hard the teams work to reach their targets. Teams work nights, weekends, and through the holidays to try to reach men when and where they are most likely to access services. One KI noted, *“When we go for outreach we never return without finishing clients who want to be circumcised on that day*. *So sometimes we finish work around 1 am*. *We work on Saturdays and Sundays*, *we do not have weekends*, *we are always on duty*.*”* Another person suggested that the provider schedules were created specifically to meet men where they most desired services. For example, miners work during the day underground, and are only able to access services at night or on weekends. *“We do moonlighting especially in areas with artisan miners*. *Many of them are busy digging for gold during the day so they come for circumcision during the night like around 8pm*.*”*

Second, money was reported as a strong motivator for achieving program targets. With productivity tied to performance based financing (PBF) incentives, money was a driver for motivating provider behavior. “*Fortunately where there is money there is hard work and where there is hard work there is more money*.*”* Extending the financial motivation to job security, another KI responded that,

“*money is the thing*, *you don’t want to run away from that*. *Of course*, *you will be motivated because you want to be on the job so you need to meet the targets*. *You need to secure your job*, *that issue of job security also motivates you*.”

Third, team members reported they were well trained and prepared to expand VMMC service delivery to reach ambitious targets. VMMC training has been expanded nationwide and integrated with other routine nurse training, more HCWs are available to rotate into VMMC duty so that sites *“always have a team here at the static site*. *We accept walk-in clients during working hours*. *There is always a team on stand-by here while others go for camping and outreach*.*”* Even without the ZAZIC support, sites are creative in engaging help when needed. “*Whenever we are overwhelmed*, *the city council people come and assist*. *We divide ourselves*, *share roles… someone is doing the counseling*, *someone is testing*, *and someone is doing the cutting*.*”*

Lastly, VMMC teams knew how to leverage their strengths for maximum productivity. For example, *“we just try to work in teams and even choose people…there are clinicians who are more experienced in these things and who have better speed so we [are a] strategical pair ourselves so that we also speed up the process while still maintaining quality*.*”* Complementing the importance of this emphasis on strategic human resource management, another KI reported. that ZAZIC roving teams who augment the service delivery teams bring welcomed vehicle support. “*We call on that support team when we are overwhelmed with numbers*, *they come and support us with a vehicle and staff*.*”* Vehicles to support service delivery, demand creation and follow-up were reported as key: the *“availability of the vehicle makes our work easier and we reach more clients*.*”*

### Ensuring patient safety

All KIs noted that the VMMC procedure is performed according to SOPs and with quality assurance efforts by team members. Even with the mandates for high numbers and the time needed to reach some outreach locations, the teams indicated that they would not conduct VMMCs if the conditions were not amenable to client safety. Outreach locations occurred in rural health centers or schools, but often were conducted in tents where clinicians may have more control over cleanliness, preparation, and VMMC service provision. However, sometimes the KI team members reported they chose not to perform VMMCs over operating in poor conditions. As this clinician notes:

*“We have avoided circumcising clients in some clinics… If you check room you might notice it is not suitable for VMMC procedures*. *When that happens we prefer to use mobile tents…if there are no tents available we go to the next clinic*. *We want to maintain patient safety*.”

Although respondents noted that they aim for procedure efficiency, the teams were all aware of the critical nature of maintaining client safety, and there are few shortcuts on procedures. Unlike other medical procedures undertaken due to need, VMMC is an elective procedure performed on healthy men and boys, making it even more important that they clients enter and leave without harm.

*“The outreach site is different*, *the facilities*, *the equipment that you use is different from static site*. *But in terms of the provision itself*, *in terms of actual surgery*, *there is no compromise*.”

### Prioritizing adolescent care

To ensure client safety, adolescents received special treatment. Teams reported working hard to finish all younger clients so they could be returned to their homes before dark. As many of these young boys were only familiar with their homesteads or foot pathways from school in the light, they were often unable to provide directions for a car or find their way at night. Therefore, the teams noted that, “*we want to finish the circumcisions early and deliver the younger clients before dark especially those who stay further away*. The teams also noted the importance of meeting an adult or guardian to ensure that wound care instructions are given to an adult. If an adult provides consent, but does not come to the clinic with the minor, the teams reported that they reach out to someone at the homestead to help ensure client wound healing. One KI responded that, *“we might get clients at a school*, *but we must leave them at their homes so that we talk to their parents or guardians on wound care and on how they can contact us if they have problems*.*”* Also, the teams work hard to confirm consent documentation of all younger clients as some younger clients may be tempted to falsify consent documentation if their guardian is traveling or disapproves. *“I think if the client is below the age of 18 it’s wise to phone the next of kin before circumcising…Calling will help to confirm signing of the consent form*.*”*

### Program challenges

#### Poor adverse event reporting

Despite emphasis from program supervisors on identification, management, and reporting of AEs, there are still few that are reported. The men are healing well and severe AEs are rare, with few long-term morbidities. However, there are several reasons for continued underreporting of AEs. First, there is still some AE misclassification. For example, one KI noted that, *“some of the moderate AEs maybe classified as mild AEs*, *yet mild AEs are not reported*.*”* This may indicate some gaps in AE training and reporting. Second, despite routine efforts to ensure that providers are complimented on their reporting of AEs as a sign of quality patient care, provider fear of AE reporting remains. There are still concerns of *“intimidation of teams when an AE occurs”* or worries about repercussions:

*“I think it is because of what the partners tell us*. *They are always saying AEs cost them lot of money* [to treat], *so staff end up afraid to report the AE*. *Sites might try to manage the AE on their own and they only report when the situation is out of hand*.”

Third, KIs commented on the volume of paperwork required for documenting moderate or severe AE which served as a disincentive to reporting.

*“You know*, *you don’t just document and send; you just have to do follow ups writing on the same document*. *You have phone calls from the head office*, *from everywhere trying to find out about the AEs and their status…the documentation can be lightened*.”

Due to these factors, it appears that many AEs are simply handled by the site teams and not reported, as revealed by this KI:

*“When AEs are being investigated at sites*, *they become like fault finding missions*. *They want to know who did what*, *what procedures were used and then what happened…endless questions*. *So due to that some sites might just decide to manage the AE*, *the client heals and the issue is resolved*.

Although the vast majority of clients heal without complication, these AE reporting weaknesses can have negative consequences on clients.

*“People just think that reporting an AE is like hanging yourself*. *That is the main issue*. *People do not want to be found at fault so they choose not to report*. *They will remain quiet about AE incidents even though this puts the clients at risk*. *Sometimes the site is not able to manage the AE and when they finally report it the AE would have gotten worse*.”

#### Prioritizing VMMC numbers over follow-up reviews

Post-operative follow-up on days 2 or 7 are important for early detection of AEs. Day 2 visit attendance is more likely as *“the client is bound to come because they don’t like the bandage on them*, *they are bound to come back*.” Although most school boys have at least one post-operative review, most men do not. “*Younger clients usually follow instructions*, *adults don’t*. *If an adult is told by a doctor to come for a review*, *they will not come back if they feel better*. *They think going back is a waste of time*.*”* Realities in the field also compromise follow-ups.

*Older clients do not come back after day 2 review*. *Some are working clients*, *some were circumcised here while visiting relatives yet they normally stay in Harare*. *Sometimes we have to call them and ask if they are OK and we just encourage them to visit the nearest VMMC site for review*. *For most day 7 reviews we usually just do them over the phone*.

Other constraints are due to the outreach locations that teams now use to reach those harder to access clients.

*“I think from my own experience these adverse events*, *these days they occur on sites that are hard to reach*. *It is sometimes a challenge to follow up on circumcised clients*. *In some areas there are no roads*, *you cannot use a car*, *you reach them on foot and that is how some clients are missed*. *At times when you finally access the area adverse events would have occurred*. *Easy to reach clients can be reviewed easily compared to those who stay in mountainous areas*. *At times we liaise with the mobilisers and ask them to do the reviews*.”

Other teams may conduct follow-up reviews but not report AEs treated in the field since they *“will say what is important is reporting the numbers done*, *what does it change to report adverse events*?*”*

#### Effects of stigma on patient follow-up

Circumcision-related stigma reduces follow-up. VMMC follow up is a standard part of VMMC service delivery and included in the VMMC consent process. However, some men may be concerned that others may find out about their circumcision status and, therefore, provide false information including contact details. One provider noted that when they contact clients to see how they are healing, the clients “*are actually surprised when we call*. *They tell us we should leave them alone’*, *they got circumcised and that is all they wanted*, *they don’t want to be followed up*.*”* To avoid follow-up, some men go to other extremes by giving false information.

*“Recently we had a client here who was served*, *and on his way out*, *he confessed that all the information he had given us was false including his name (laughs)*. *He said he was wary that we would tell everyone that he had been circumcised*.”

Other men, or even boys, ask to be dropped off by the VMMC vehicle far from their homes to avoid being associated with the branded car.

*“Sometimes after circumcision they ask the driver to drop them off away from their homes*. *Such clients are the ones who usually give false information*. *They do not want VMMC teams to visit them at their home*, *they prefer coming to the clinic when they have problems*. *They will tell you they stay in the west then in fact they stay in the east*.”

Therefore, even if the providers wanted to conduct follow-up, they couldn’t. *“If they gave you false information and they see the VMMC vehicle in the area they will hide*. *How will you find them*? *You can’t*.*”*

### The Trade-offs in quantity over quality

#### Pressure to reach targets encourages poor provider care

From provider reports it appears that increasing targets may threaten service quality. One provider noted, *“There should be less pressure on the targets so that team give quality service*. *If teams are pressurised*, *they will do short cuts*. *Also*, *there are sites which are short staffed yet they continue receiving pressure to meet targets*.*”* Reaching high numbers also appears inconsistent with the reported VMMC saturation in many districts. As the number of circumcised men goes up, there are fewer men to target. Finding enough men to meet the expected productivity can result in temptation to cheat. One KI commented that, *“If targets are ‘out of this world’ this might force people to do bad things*. *Remember the VMMC programme has been running for more than 6 or 7 years now in the same districts and clients are being circumcised*. *We should also consider that numbers are going down*.*”* As one respondent summed up in response to meeting the targets when demand was actually low, *“people being people*, *they can just manipulate the system*, *trust me*.*”* In terms of follow-up, several KIs mentioned that teams prioritized VMMCs over reviews, potentially leading to missed AEs as VMMC teams *“won’t do day 7 reviews because they will be focusing on going to other areas to circumcise so that they meet their targets*.*”*

#### Pre- and post-operative counseling is shortened or combined

Multiple KIs noted that pre- and post-operative counseling is reduced to save time, allowing for more VMMCs with less overall time. *“What is sometimes done is combining individual counselling*, *examination and surgery at the same time*.*”* However, by trying to shorten and combine information, the knowledge may not be communicated accurately. *“At times you find the whole group* [of counseled clients] *will give you the same incorrect information*. *So that means there were some mistakes which were done during the counseling*.*”* As counseling is the key to early AE identification and management, one provider was particularly concerned with this practice. One noted that, “*the only thing that prevents AEs is educating the client on wound care*. *Clients will follow instructions if given enough information*. *The challenge is most of the time service providers do not have enough time to educate the client*.*”*

#### Documentation suffers

With pressure on achieving targets and the pace of VMMC implementation, VMMC documentation may suffer, leading to discrepancies between reported VMMCs in outreach and static sites. One KI revealed that, *“a team may go for outreach*, *they circumcise and when they come…*. *you should see how people behaving after an outreach…*. *they will be tired*, *everything will be haphazard and so forth*. *The discrepancies are because client forms get lost*.*”* Data quality audits also find incomplete data that participants linked to the emphasis on the numbers. For example, *“you might find a CR [client record] Form with blank spaces and missing information because the one who was to complete the section was in a rush to meet targets*.*”* These documentation weaknesses also lead to differences between VMMCs reported and VMMCs verified, raising concerns on payment based on VMMC performance. For example, clients may all be placed in the register and not removed if they are found ineligible as, “*on registration you realize there are [VMMC] contraindications and we don’t circumcise the client*. *That client is already captured in the register and might be counted as a circumcised client*.*”* These inconsistencies were reported to be reflected in stock records where VMMC *“kits are not recorded on site stock cards…[roving] teams just use those kits*, *they don’t record them*.*”*

#### Client recovery time is shortened

Apparent shortcuts in recovery were also reported. The earlier clients have ample time for rest while, to get clients home, those last served may be transported before early signs of complication can be noted. Although each client is supposed to rest for at least 20 minutes after the VMMC and be closely monitored over this period, “*sometimes post-op observations are not taken*. *The first client to be circumcised and the last client do not have the same amount for resting after the procedure*.*”*

Also, in the push to bring services close to the clients, some of the sites selected do not have ample space for patient rest after VMMC. The clinicians must choose to prioritize the procedure over other patient care priorities. For example, one KI said that:

*“some of our sites don’t have enough room…or maybe when we are on outreach…remember we want our clients to recover on a bed*. *If we have got two beds*, *we will use them for the actual procedure and just ask circumcised clients to sit and relax on a chair*.”

#### Not all guidelines are followed for the VMMC procedure

Although all providers noted that safety was prioritized, there are weaknesses in the actual VMMC procedure. First, as one provider noted, *at some point teams might rush and by-pass some procedures*. *This might result in infections*. *Screening might not be done properly and this results in compromised procedures*.*”* As the pre-op assessment is supposed to be conducted for VMMC eligibility, a poor assessment, or no assessment, may lead to boys or men with contraindications (phimosis, hemophilia, keloids) being circumcised. Second, all hygiene or infection prevention measures may not be performed.

*“You are supposed to clean 3 times using betadine antiseptic (uses hand to show how the cleaning is supposed to be done) but maybe because of pressure a provider may only clean once and then quickly circumcise*. *Also after cleaning with betadine you are supposed to wait for 2 minutes for the betadine to work so maybe because of pressure one might rush to circumcise without waiting for the betadine to work…What happens is that some providers ask the client to lie on the bed and they then withdraw the medicine before cleaning the area*. *If you clean the area after having drawn the medicine the temptation is to inject without waiting for the 2 minutes for the betadine to work*. *Such acts of not following procedures compromise the quality*.”

## Discussion

This qualitative study within a routine VMMC program operating at scale in Zimbabwe found that VMMC teams work tirelessly and creatively to achieve time-bound, donor-driven, ambitious VMMC targets. In the environment of worsening economic outlook and severe constraints on the electrical, water, and cell networks, the VMMC teams persevere. VMMC teams employ demand creation innovations, rotate teams, work long hours, and adapt their program strategies to reach men in diverse settings. However, reaching these productivity levels may compromise the quality of VMMC service delivery in these remote, rural, and/or low-resource service delivery settings. To achieve ambitious VMMC targets, teams at times may cut corners in counseling, recovery, and VMMC hygiene procedures to shave time off the VMMC process and complete more VMMCs. The drive to achieve targets is largely motivated by money. It is unsurprising that financial incentives were a motivator to VMMC productivity all along the service delivery continuum–from providers to support staff, and government leadership all benefit [24]. Patients also seem motivated by the reduced risk of HIV acquisition and other demand creation strategies. However, in pursuit of targets, health workers may compromise quality of VMMC services, creating an unacceptable trade-off between productivity and patient safety. Several key outcomes from this study can be used to inform further scale up of VMMC in Zimbabwe and the region.

First, despite low AE rates in the ZAZIC program that suggest overall patient safety [27–29], our results indicate that the drive for target achievement leaves quality gaps throughout the cascade of VMMC service delivery. Most KIs noted weaknesses in counseling sessions: both pre-and post-operative counseling may be shortened or group and individual sessions may be combined. These shortcomings in care put clients at risk since information provided during pre-operative counselling sessions contributes to the best outcomes in VMMC care, especially for adolescents [30–32]. Rushed or combined group and individual counseling sessions may result in critical information being omitted by providers or lack of understanding of critical post-operative instructions, either of which leads to poor wound care practices and occurrence of AEs. Although it appears that adolescents receive additional wound care instruction and are prioritized by VMMC teams to help ensure their understanding and safeguard the health of younger clients, other clients should not be left short-changed. Adults also require high quality pre- and post-operative counseling to ensure consent verification, VMMC procedure understanding, wound care instructions, and sexual abstinence during the healing process as required by VMMC standard operating procedures.

Second, several critical weaknesses were noted in the surgical procedure, itself, in response to the need for faster VMMC procedures. Inadequate provider scrubbing, abbreviated hygiene procedures to ensure client preparation, and shortened client recovery time before discharge were all mentioned as shortcuts in the VMMC procedure. Although similar findings of providers scoring low in infection control procedures, omitting post-operative observations, or not maintaining sterile surgical fields were considered lack of training or skill set deterioration in a three country study [33], the KI remarks from this study likely do not reflect lack of skill or knowledge. ZAZIC’s VMMC clinicians are certified to provide standardized services, receive continuous mentorship, and are monitored by both internal and external quality assurance assessments. Omissions in VMMC procedures reported in this study are reflective of efforts by clinicians to circumcise more clients within shorter time periods. As VMMC teams continue to work tirelessly to meet quantity and quality expectations, prolonged hard work by teams may ultimately lead to fatigue and compromise quality of VMMC services. In response, additional spot-checks, unplanned observational visits, or strengthened routine monitoring of providers are needed to assure adherence to standard operating procedures, adjust VMMC service delivery timing to avoid rushing to meet demand within expected service delivery windows, and better equip outreach facilities with recovery spaces.

Third, VMMC teams enhanced client safety by prioritizing follow up of adolescent boys and providing postoperative care in the presence of parents or guardians. This effort was in direct response to ZAZIC [27–29] and previous research [34–36] indicating that adolescent boys are at higher risk for infections due to poor post-operative care. However, added attention on younger clients may diminish focus on adult men. KIs reported that although most adults returned for post-operative review on day 2 for bandage removal, most never attended nor were traced for subsequent reviews on day 7 when risk of AEs are higher [28,37,38]. Providers attributed the lack of later reviews on competing priorities that reward productivity over post-operative reviews. This leads to VMMC teams concentrating on getting new clients over confirming healing of those already circumcised. By not fulfilling recommended post-operative reviews, VMMC programs rely on men’s appropriate and timely health seeking behavior in the event of complication. Men, themselves, also appear to ignore educational counseling and defer to their own assessment of healthy healing. To fill the gap left from decreased attention to in-person follow-up, a recent mobile health (mHealth) study in Zimbabwe found that men were capable of healing safely at home when accompanied by daily, two-way texting with providers to ensure swift identification of, and review for, potential AEs [39]. Additional study of alternative follow-up methods (texting or incentives for attendance) and expansion of follow-up review capacity at rural health centers is warranted to safeguard VMMC patient safety.

Lastly, pervasive AE underreporting distorts perception of the simplicity and speed of the VMMC procedure and curtails resources needed to provide quality care [29]. This study illuminated additional reasons for AE underreporting: providers feared shouldering blame for AEs, perceived the AE paperwork and reporting burden as formidable, and were confronted by negative responses from supervisors when AEs were reported. In response, KIs stated that providers or rural health teams manage AEs locally without reporting, putting patients at risk if management is inappropriate or requires higher levels of care. Conversely, the findings also point to overall patient safety within the VMMC program as AE rates remain low and severe disabilities resulting from VMMC are rare. To address this persistent problem, ZAZIC teams are working to change the culture of reporting to reward AE documentation as a sign of quality program delivery, encouraging fault free reporting while reducing duplication in AE reporting requirements.

Perhaps most interesting from this study was the finding on VMMC-related stigma, an issue largely absent in the literature. One study from Zimbabwe found that uncircumcised men identified stigma from community and even family members as a hindrance to accessing circumcision [40]. However, KIs reported stigma post-operative in the VMMC care context. Although demand creation teams are able to find men willing to champion the VMMC program and encourage others to seek VMMC services, this study found that although men wanted to receive VMMC and were successfully circumcised, many of those same men did not want to be identified as circumcised. Almost all VMMC teams reported that they frequently encountered men who did not want VMMC teams to conduct follow-up reviews or arrive in branded cars at their homes or workplaces. Respondents noted that some men provided inaccurate names or homestead locations to prevent follow-up communication and, instead, preferred to contact the VMMC team only if they needed. The issue of stigma as a barrier to VMMC follow-up and post-operative care seeking behavior warrants future study.

Our findings on balancing the quality versus quantity of VMMC service delivery in routine settings are in line with several recent studies from sub-Saharan Africa that found similar unintended consequences of the drive for VMMC program saturation. In a quantitative study from South Africa [15], notable challenges in VMMC program quality such as poor monitoring of AEs, lack of adequate provider training in expansion sites, and sub-optimal supervision were likely related to donor-driven and Ministry-required VMMC program expansion within a short timeframe. Similarly, a qualitative study of the unintended consequences of VMMC target achievement on the quality of VMMC care received by adolescents in Kenya found misleading demand creation strategies, long wait times for clients, exhausted clinic workers, and lack of adherence to VMMC best practices [41]. They also suggest more input from local VMMC providers, mobilizers, and implementing partners should be considered for target setting and call for strengthened adherence to standards for responsible conduct of VMMC. Lastly, in the push to reach all potential VMMC clients at a rapid pace, another study identified issues in consent verification among adolescent Kenyan VMMC clients [42], further calling for reconsideration of the direct linkage between VMMC target achievement to program success.

## Limitations

Hesitation among potential respondents on being audio recorded could have limited full disclosure of VMMC quality issues; however, interviewer reminders on confidentiality eased tension and made respondents more at ease. In addition, the sensitive nature of the questions asked which, in a way, requested clinicians to self-report certain questionable practices may have limited full disclosure by participants. Convenience sampling was used to select participants for KIIs and the modest number of KIIs may limit generalization of study findings. Sites received different support from ZAZIC (vehicles, demand creation support, additional providers) which could have influenced their responses. Finally, entering, coding and analyzing of qualitative data by one external researcher to protect KI confidentiality may have led to unintentional bias as the codes were not cross validated.

## Conclusions

Although current risk to patient safety for this voluntary procedure appears minimal, client safety must be prioritized over productivity. Realities and risks should be carefully considered by donors and Ministries to not unrealistically or blindly require programs to do more with less. In addition, VMMC programs should focus on components of service delivery such as provider perception of AE reporting and the reporting burden. VMMC teams should be consulted and local program conditions and constraints closely evaluated when new VMMC targets, policies and standards of practice are crafted. Independent active surveillance measures such as tandem reviews should also be employed as a reference for program quality. The balance of quality and quantity has to be maintained by providing adequate resources across the VMMC service delivery spectrum in support of demand creation, provider training, and adequate transportation to conduct post-operative reviews. Additional resources for VMMC procedures and review is more relevant now as maturing VMMC programs must reach further into remote or rural locations and increase demand creation efforts for those harder to reach men to attain program saturation. Importantly, a more participatory approach to target and UE setting that includes input from implementing partners, CDC and central funding sources could help balance continued high VMMC performance alongside strengthened program quality.

## Supporting information

S1 File Key informant interview: District medical officer/site focal person. (DOCX)
