# Supplementary material for: Balancing competing priorities: Quantity versus quality within a routine, voluntary medical male circumcision program operating at scale in Zimbabwe
Source: PLoS One. 2020 Oct 13;15(10):e0240425. doi: 10.1371/journal.pone.0240425 (PMC7553309; doi:10.1371/journal.pone.0240425)
Supplement: S1 File — (DOCX) [file pone.0240425.s001.docx]

Key informant interview: District Medical Officer/Site focal person:

**FIRST:** Interviewer MUST verify that informed consent is signed by the interviewee

Verification of informed consent? CIRCLE ONE: YES NO

General introduction:

Hello, my name is ____. Thank you for agreeing to talk to me/us today. We are here to talk with you as part of the ZAZIC organization that implements the national male circumcision program in this district. As District Medical Officer, your experience and thoughts about the male circumcision (VMMC) program are critical to our understanding of the strengths and weaknesses of the program. We would like to thank you for agreeing to participate in this interview. Today, we would like to talk with you briefly about the VMMC program, and specifically about reaching the facility and district targets. I/we will be asking you some questions which you are free to answer in any way you wish. You may also choose not to answer any question. We encourage you to feel free to say anything concerning the topic of discussion. If a question is unclear to you, you can ask me to explain it. Your participation is voluntary and confidential.

Whatever you tell us will be treated with utmost confidentiality. The information will only be used for the purposes of this program evaluation.

I would like to request that you allow me to record our discussion so that I don’t miss anything. Your voice will not be heard by anyone other than our I-TECH study transcriber. Your name and district will not be recorded and will not appear on the transcription. The tapes will be destroyed after we have prepared our transcripts.

Is it okay if I tape record our discussion? YES NO*

*If NO, STOP INTERVIEW HERE AND THANK THE RESPONDENT

1. **Program success***Thank you for speaking with me today. I would like to start by asking you about what you think are the major successes of the VMMC program here?*

Probes

- What are the successes for the community?
- For healthcare workers?
- For the clinic?

1. **Program weaknesses***Now we would like to discuss the program challenges. What do you think are the major weaknesses of the VMMC program here?*

Probes

- Anything else?

1. **Achieving targets**

*We are interested in learning more about your high performance at XX site. It is quite productive. Tell me about how you achieve your district targets.*

Probes

- - How do you increase demand?
  - What strategies do teams take to meet demand?
  - When demand is high, how do teams adapt to perform the VMMCs?
  - What are some of the ways that the teams innovate to increase productivity? (Probe: Nights? Weekends? Long shifts?)

1. Team stress

*We are also looking to gain information into the stress that your teams might feel to reach the high targets. How do teams handle the high work load?*

Probes

- How does the work load vary by day?
- How many days a week do staff stay late or work outside of normal hours to complete the day’s VMMCs?
- What motivates them to work so hard?

1. Maintaining safety

*Now lets talk about patient safety Please remember that we are not asking you to identify specific sites or providers, but just report on your observations and experiences. Tell me how you maintain patient safety with so many procedures?*

Probe:

- What modifications (i.e., the original SOPs, guidelines or initial training) do they need to make to adjust to the realities of the field?
- What types of outreach settings have you avoided to maintain patient safety?
- What do teams do when they are concerned about field conditions for hygiene or safety?

1. AEs

*Now lets talk about adverse events. The reported adverse event rate here is low, as with many of the sites in Zimbabwe. Yet, our recent ZAZIC quality assurance activity found AE rates to be higher between 1-8%. Why do you think site reported AEs are lower? Again, we are not asking you to identify specific sites or providers, but just report on your observations and experiences.*

Probe:

- How are adverse events documented in the field? Static and outreach
- How might moderate or severe AEs not be reported?
- What pressure or fear is there to not report AE?
- What are your concerns in terms of prevention, identification and management of AEs?
- How can we improve AE reporting?

1. Follow-up

*Let’s talk about follow-up. In your estimation, what percent of men do you think attend the Day 2 visit in static sites? Outreach sites? What about for Day 7 in static sites? Day 7 in outreach? Do these estimates change depending on client age?*

Probe:

- What do you think contributes to lack of follow-up?
- How do you help ensure adherence to the 2-Day and 7-day follow-up visits in outreach settings? In static settings?
- What are your concerns with adherence to these visits?
- How can we improve follow-up in rural areas?

1. Quality/Quantity balance

*Let’s talk about the balance between productivity and safety. How might VMMC program quality be compromised to increase productivity and reach targets?*

Probe:

- What corners may be cut to speed VMMC service delivery? (Examples: less time on counseling, prepping the surgical area, procedure, recovery).
- How might serious AEs be missed due to the push for numbers?
- How might teams inflate numbers to show increased productivity?
- What steps can be taken to ensure that reported VMMCs numbers reflect true VMMCs?

1. *Lastly, tell us about the overall quality of service provision at this site?*

Probe:

- What concerns would you have sending a loved one for VMMC at this site?

1. *What final thoughts about the male circumcision program would you like to share?*

END OF SESSION

“*Now we have come to the end of our discussion.* *Thank you for speaking with me today. This was a really valuable conversation, and we greatly appreciate your time.”*

## Key informant interview: ZAZIC clinician

**FIRST:** Interviewer MUST verify that informed consent is signed by the interviewee

Verification of informed consent? CIRCLE ONE: YES NO

General introduction:

Hello, my name is ____. Thank you for agreeing to talk to me/us today. We’re here to talk with you as part of the ZAZIC organization that implements the national male circumcision program in this district. As a ZAZIC clinician, your experience and thoughts about the male circumcision (VMMC) program are critical to our understanding of the strengths and weaknesses of the program. We would like to thank you for agreeing to participate in this interview. Today, we would like to talk with you briefly about the VMMC program, and specifically about your role in helping reach the facility and district targets. I/we will be asking you some questions which you are free to answer in any way you wish. You may also choose not to answer any question. We encourage you to feel free to say anything concerning the topic of discussion. If a question is unclear to you, you can ask me to explain it. Your participation is voluntary and confidential. Only I-TECH study team will review transcripts. Your supervisor will not be able to link your name with this interview transcript.

Whatever you tell us will be treated with utmost confidentiality. The information will only be used for the purposes of this program evaluation.

I would like to request that you allow me to record our discussion so that I don’t miss anything. Your voice will not be heard by anyone other than our I-TECH study transcriber Your name and organization will not be recorded and will not appear on the transcription. The tapes will be destroyed after we have prepared our transcripts.

Is it okay if I tape record our discussion? YES NO*

*If NO, STOP INTERVIEW HERE AND THANK THE RESPONDENT

1. Role in VMMC program

*To begin, can you please tell me about your role in ZAZIC?*

Probes

- Do you perform VMMC? How many, about?
- Do you conduct VMMC supervision? For how many teams/districts?
- Do you train others in VMMC? For how many teams/districts?

1. **Program success***Now we would like to discuss the successes of the ZAZIC program. What do you think are the major successes of the VMMC program overall?*

Probes

- What are the successes for the community?
- For healthcare workers?
- For the clinics?
- For the ZAZIC teams, specifically?

1. **Program weaknesses***Now we would like to discuss the overall program challenges. What do you think are the major weaknesses of the VMMC program overall?*

Probes

- What are the challenges specifically for the ZAZIC roving teams?

1. Achieving targets:

*We are interested in learning more about your high roving team performances. They are quite productive and really aid the outreach and static sites. Tell me about how you achieve your team targets.*

Probes

- - How is demand generated before the roving team arrives??
  - What strategies do roving teams take to meet high demand?
  - When demand is high, how do roving teams adapt to perform the VMMCs?
  - What are some of the ways that the roving teams innovate to increase productivity? (Probe: Nights? Weekends? Long shifts?)
  - What motivates them to work so hard?
  - What are the perks of being part of the roving team?

1. Team stress

*We are also looking to gain information into the stress that your roving teams might feel to reach the high targets. How do roving teams handle the high work load? We are not asking you to identify specific sites or providers, but just report on your observations and experiences.*

Probes

- How does your team manage with the long days of travel?
- How does the team adapt to the work hours?

1. Maintaining safety

*Now lets talk about patient safety. We are not asking you to identify specific sites or providers, but just report on your observations and experiences. Tell me how you maintain patient safety with so many procedures?*

Probe:

- What modifications (to SOPs, guidelines, training) do roving teams make to adjust to the realities of the field?
- What types of outreach settings have you avoided to maintain patient safety?
- What do teams do when they are concerned about field conditions for hygiene or safety?

1. AEs

*Now lets talk about adverse events. We are interested in your observations and experiences, not specific people or places. The ZAZIC overall adverse event rate here is low, for both partners. Yet, our recent ZAZIC quality assurance activity found AE rates between 1-8%. Why do you think that is?*

Probe:

- What are your concerns in terms of prevention, identification and management of AEs?
- How are adverse events documented in the field?
- How are adverse events recorded or reported after the outreach teams leave?
- How might moderate or severe AEs not be reported?
- What pressure is there to not report AEs?
- How can we improve patient outcomes in this environment?

1. Follow-up

What about follow-up? What plans are made for the Day 2 and Day 7 follow-up visits?

Probe:

- How do you help ensure adherence to the 2-Day and 7-day follow-up visits?
- How does follow-up differ by location (static/outreach?)
- How does follow-up differ depending on MoHCC staff?
- What are your concerns with adherence to these visits?
- How can we improve follow-up in rural areas?

1. Quality/Quantity balance

*Lastly, let’s talk about the balance between productivity and safety. How might VMMC program quality be compromised to increase productivity and reach targets?*

Probe:

- What corners may be cut to speed VMMC service delivery? (Examples: less time on counseling, prepping the surgical area, procedure, recovery).
- How might serious AEs be missed due to the push for numbers?
- How might teams inflate numbers to show increased productivity?
- What steps can be taken to ensure that reported VMMCs numbers reflect true VMMCs?

1. *What final thoughts about the male circumcision program would you like to share?*

END OF SESSION “*Now we have come to the end of our discussion.* *Thank you for speaking with me today. This was a really valuable conversation, and we greatly appreciate your time.”*
